# Sex differences in psychological distress and its risk factors among US adult Black and White immigrants, NHIS 2005–2018

**DOI:** 10.1038/s41598-026-45360-0

**Published:** 2026-03-25

**Authors:** David Adzrago, Maryam Elhabashy, Faustine Williams

**Affiliations:** Division of Intramural Research, National Institute On Minority Health and Health Disparities, National Institutes of Health, 11545 Rockville Pike, Rockville, MD 20852 USA

**Keywords:** Mental health, Sex and gender disparities, Anxiety, Depression, Immigrant health, Anxiety, Depression, Risk factors, Public health

## Abstract

Limited research explores sex differences in psychological distress and its risk factors across minority communities, especially immigrant populations. This study aimed to estimate the prevalence of psychological distress and its sociodemographic, biological, behavioral, and clinical risk factors among male and female Black and White immigrants. Drawing from the 2005–2018 National Health Interview Surveys data, a sample of male (n = 21,082) and female (n = 24,984) adult Black and White immigrants (n = 46,066) was analyzed using weighted multivariable logistic regression models. In terms of biological factors, older age (i.e., ≥ 65 vs. 18–25 years) was significantly associated with lower odds of psychological distress for males and females. Being overweight was also significantly associated with lower odds of psychological distress factor for males, while being overweight or obese was significantly associated with higher odds of psychological distress for females. For behavioral factors, alcohol drinking (current and former) and smoking (current and former) were significantly associated with higher likelihood of psychological distress for both groups, but the effect sizes for drinking were higher for males while those for smoking were higher for females. With clinical factors, having one or more chronic diseases was associated with higher odds of experiencing psychological distress, especially for females. More sex-specific considerations should be addressed in mental health research and intervention to meet the needs of male and female immigrants.

**Supplementary Information:**

The online version contains supplementary material available at 10.1038/s41598-026-45360-0.

## Introduction

A growing body of research has investigated both the causes and the comorbidities associated with psychological distress, a condition characterized by an emotional state of suffering predicated on symptoms of stress, anxiety, and depression which often coexist^1,2^. Psychological distress has not only been linked to unhealthy behaviors such as poor diet, physical inactivity, smoking, and alcohol intake, but also to temporary and permanent health problems ranging from depression and anxiety to arthritis, lung disease, and cardiovascular disease^3,4^. Robust associations between psychological distress and mortality have been reported among the United States (US) adult population^5^. More recent research has indicated that psychological distress is more common among women than among men^6,7^. This may present unique challenges in the spheres of health disparities, especially since rates of psychological distress among US adults increased three-fold between March 2020 and September 2022^8^.

Few studies have examined which predictors may contribute more significantly to psychological distress within or across certain subpopulations, especially among Black and White immigrant populations. Previous research found that compared to US-born individuals, immigrants experience higher prevalence of psychological distress as length of residence in the US increases^9^. This is consistent with acculturative stress theory, which describes the tendency of immigrants to experience unique stressors due to their immigration status and the processes associated with navigating life in a new home country^10,11^. Existing research yields mixed results with regards to acculturative stress by sex. While some conclude that female immigrants are at greater risk of acculturative stress and psychological distress^12–14^, others report that acculturative stress has stronger associations with mental health symptoms among male immigrants compared to female immigrants^15,16^. Inconsistent reports of sex differences in acculturative stress may have implications for psychological distress prevalence and risk factors among male and female immigrants. Furthermore, while existing literature reports inconsistencies in immigrant population accounts of acculturative stress and psychological distress, they rarely examine Black and White immigrant populations, who remain disproportionately understudied despite their contributions to US population growth^17,18^. This represents an important research gap which can result in the homogenization of immigrant populations and the obscuring of important within- and between- group differences.

Recent evidence underscores the importance of disaggregating immigrant populations by race and nativity. Among Black or African American adults, US-born individuals experience higher prevalence of moderate-severe psychological distress compared to their non-US-born counterparts, with higher likelihoods of psychological distress being observed among individuals with younger age, divorce or separation, unemployment, lower educational attainment, lack of health insurance, and alcohol use^19^. These findings suggest that the healthy immigrant effect, wherein immigrants initially exhibit better health outcomes than their native-born counterparts, may operate differently across racial groups and erode over time due to exposure to discrimination and socioeconomic stressors^20,21^. Additionally, psychological distress prevalence and risk factor profiles differ meaningfully between Black and White immigrant populations, with more pronounced behavioral risks observed among Black immigrants, despite White immigrants experiencing a higher prevalence of psychological distress^22^. Within-group and between-group heterogeneity highlights the need for research that moves beyond treating immigrants or racial groups as monolithic populations^19,23^.

Theoretical frameworks and extant psychological distress research suggest that sociodemographic (e.g., age, sex, employment and educational status) and behavioral (e.g., smoking, alcohol use) factors. Specifically, acculturative stress theory and the multiple minority stress model posit that individuals holding multiple marginalized identities may experience compounded stressors that elevate their risk for psychological distress^24–27^. The behavioral factors such as smoking, alcohol use, lack of physical activity, as well as clinical factors such as chronic diseases and body mass index (BMI) have been associated with psychological distress in nationally representative samples^28–31^.

This study had two aims: (1) to estimate the prevalence of psychological distress and (2) to assess its risk factors among male and female adult Black and White immigrants. Specifically, sex-specific risk factors for psychological distress among adult Black and White immigrants in the US. Consequently, the findings can help develop public health strategies tailored to the needs of male and female immigrants and better understand the experiences and risk factors of individuals within these understudied immigrant populations.

## Methods

### Data sources and sample

This study relied on data from the 2005–2018 National Health Interview Survey (NHIS), a nationally representative cross-sectional survey conducted annually within the US civilian population who are noninstitutionalized and reside within the 50 states and the District of Columbia^32–34^. NHIS is funded and conducted by the National Center for Health Statistics (NCHS) at the Centers for Disease Control and Prevention (CDC). By partitioning the US into several nested levels of strata and clusters, a random sample of dwelling units and participants are selected (i.e., stratified complex clustered sampling)^32,34^. The NHIS collects information on sociodemographic characteristics (e.g., age, sex, nativity, race/ethnicity), mental and physical health conditions, and health behaviors (e.g., physical activity, substance use) among children aged ≤ 17 years and adults aged ≥ 18 years^32,34^. For this study, analysis was based on a pooled sample of 200,693 immigrant adults (i.e., individuals not born in the US). Data collected beyond 2018 were not included in the current analysis due to content and structural changes to the NHIS, which was redesigned in 2019 and thus rendered incompatible with earlier NHIS data. We also restricted the analysis to Black and White immigrant adults (n = 55,751) who identified as male (n = 25,072) or female (n = 30,679). We then conducted complete case analyses based on psychological distress among 46,066 immigrant adults who identified as male (n = 21,082) or female (n = 24,984) (see Supplementary Fig. S1 for detailed information on participants’ inclusion and exclusion criteria). The NHIS documents (e.g., questionnaire) and datasets are deidentified, publicly available in the CDC database repository and can be accessed by all researchers, policymakers, and other data users. These public-use datasets and measures are also available at the Integrated Public Use Microdata Series (IPUMS) NHIS database, which is used to combine and harmonize data across survey years.

### Measures

#### Psychological distress status

The Kessler Psychological Distress Scale (K6) was used to ascertain psychological distress status among the participants. The K6 is a widely used scale for assessing overall psychological distress in the NHIS dataset^35–37^. Using a 5-point Likert-type rating scale ranging from 0 (none of the time) to 4 (all of the time), participants self-reported responses to questions asking how often they felt sad, nervous, restless, hopeless, worthless, or that everything was an effort within the past 30 days^38^. Total scores ranged from 0 to 24, with higher scores indicating higher psychological distress^35,38,39^. If participants’ scores were ≥ 5, this indicated moderate to severe psychological distress; scores < 5 indicated no to mild psychological distress^35,38,39^. These psychological distress scores and dichotomization have been established to warrant further clinical attention and treatment^22,35,40–46^. Particularly, the ≥ 5 cutoff has been identified as an optimal lower threshold for detecting moderate mental distress based on mental health treatment need and utilization^40^. This dichotomization between clinical (≥ 5) and non-clinical (< 5) populations has been replicated across diverse samples and is widely used in epidemiological research to capture individuals experiencing psychological distress that may benefit from mental health intervention^22,29,35,40–48^.

#### Predictors of moderate-severe psychological distress

For this study, predictors for moderate-severe psychological distress included sociodemographic characteristics, behavioral, anthropometric, and clinical factors. The NHIS questions used to assess these factors with their detailed descriptions and recategorizations are presented in Supplementary Table S1. Sociodemographic characteristics included race (Black or White), age (18–25, 26–34, 35–44, 45–54, 55–64, or ≥ 65 years), marital status (divorced, separated, widowed, married/living with partner, single/never married), employment status (employed or unemployed), poverty status (below poverty threshold or at/above poverty threshold), level of education (less than high school, high school, some college, or ≥ college degree), health insurance status (insured or uninsured), and region of residence (Northeast, North Central/Midwest, South, or West), and acculturation/length of stay in the US (< 10 years or ≥ 10 years).

The health behaviors that were analyzed included physical activity status (inactive/insufficiently active or physically active), alcohol drinking status (never, former, or current drinker), and cigarette smoking status (never, former, or current smoker). The 2018 Physical Activity Guidelines for Americans was used to define physical activity status (HHS, 2018). Participants were classified as inactive/insufficiently active if they did not meet moderate and vigorous leisure-time physical activity requirements (goal: 150 min per week of moderate activity, 75 min per week of vigorous activity, or an equivalent combination of the two); those who did meet the requirements were classified as physically active. Alcohol drinking statuses were defined as non-users/lifetime abstainers or never users (fewer than 12 drinks in their lifetime), former users (at least 12 drinks in any year of their lifetime, but no drinks in the past year), and current users (1 to 11 or more drinks in the past year). Smoking statuses were defined as never users (never smoked 100 cigarettes in their lifetime), former users (ever smoked 100 cigarettes in their lifetime but currently do not smoke cigarettes), and current users (ever smoked 100 cigarettes in their lifetime and currently smoke cigarettes). Body mass index (BMI status) (underweight/normal [BMI < 25], overweight [BMI ≥ 25 and BMI < 30], or obese [BMI ≥ 30]) was assessed as an anthropometric factor. Additionally, multiple chronic diseases (none, 1 to 2 diseases, or 3 or more diseases) were analyzed as the clinical risk factor.

### Statistical analysis

 Sampling weights were used to produce accurate and nationally representative population estimates, accounting for the survey’s multistage, complex sampling design and nonresponses^49^. The sampling weights were derived from person-level weights indicating the number of population units that a sampled unit represents. The pooled sampling weights were calibrated or adjusted (i.e., multi-year weight adjustment) by dividing the pooled weights by total survey years to compute average population estimates over 2005–2018 survey years. As displayed in Table 1, we first computed unweighted frequencies with their corresponding weighted percentages, using descriptive and bivariate statistics stratified by sex (male and female immigrants). Rao-Scott χ^2^ tests were used for the bivariate statistics to test sex-differences in the prevalence of moderate-severe psychological distress based on the various predictors. We also used two multivariable logistic regression models to examine the associations between psychological distress and the predictors (Table 2). Model 1 assessed the association among male immigrants and Model 2 assessed the association among female immigrants. Prior to conducting the stratified logistic regression analysis, we examined the interactions between sex and each of the factors (Supplementary Table S2). We further examined the interaction effects for the significant interactions by estimating the average predicted probabilities using margins command in Stata. We presented the predicted probabilities in graphs with margins plots. The level of statistical significance was set as a two-tailed p < 0.05. Odds ratios with 95% confidence intervals (95% CIs) were reported as estimates for the logistic regression models. Statistical analyses for this study were conducted using STATA version 18.0.

**Table 1:** Psychological distress prevalence by sociodemographic, behavioral, and clinical characteristics of male and female us immigrant adults (n = 46,066).

	Male		p-value	Female		p-value
	Total sample	Psychological distress (Yes)		Total sample	Psychological distress (Yes)	
	N (%)	n (%)		N (%)	n (%)	
Overall	21,082	3,280 (15.58)		24,984	5,715 (22.68)	
Race			0.034			**0.022**
White immigrant	18,347 (86.72)	2,877 (15.83)		21,780 (86.92)	5,075 (22.98)	
Black immigrant	2,735 (13.28)	403 (13.98)		3,204 (13.08)	640 (20.65)	
Age			< 0.001			< 0.001
18–25 years old	2,097 (10.10)	320 (15.92)		2,084 (8.59)	412 (20.56)	
26–34 years old	4,198 (20.53)	573 (13.87)		4,895 (18.84)	946 (19.49)	
35–44 years old	5,160 (23.37)	729 (14.20)		5,948 (22.15)	1,262 (20.79)	
45–54 years old	4,118 (19.05)	683 (15.87)		4,467 (17.55)	1,119 (24.33)	
55–64 years old	2,644 (12.92)	459 (17.24)		3,244 (13.51)	899 (26.69)	
≥ 65 years old	2,865 (14.03)	516 (18.22)		4,346 (19.36)	1,077 (24.57)	
Acculturation			0.005			< 0.001
Less than 10 years	4,649 (22.03)	646 (14.07)		4,781 (18.98)	935 (19.68)	
10 years or more	16,433 (77.97)	2,634 (16.01)		20,203 (81.02)	4,780 (23.38)	
Marital status			< 0.001			< 0.001
Divorced	1,952 (9.70)	391 (20.37)		3,128 (12.79)	893 (28.89)	
Widowed	583 (2.81)	117 (20.35)		2,387 (10.45)	667 (27.45)	
Separated	871 (3.78)	195 (21.21)		1,705 (6.00)	538 (30.44)	
Married/living with partner	13,255 (62.11)	1,838 (13.82)		13,505 (53.78)	2,551 (18.60)	
Single/never married	4,421 (21.60)	739 (16.88)		4,259 (16.98)	1,066 (25.21)	
Region of residence			0.014			< 0.001
Northeast	3,961 (20.72)	644 (15.41)		5,411 (23.66)	1,342 (23.98)	
North Central/Midwest	2,336 (12.32)	403 (17.51)		2,537 (11.45)	590 (23.16)	
South	7,750 (37.03)	1,142 (14.48)		8,930 (35.73)	1,882 (20.57)	
West	7,035 (29.93)	1,091 (16.28)		8,106 (29.16)	1,901 (24.01)	
Employment status			< 0.001			< 0.001
Employed	15,880 (75.08)	1,947 (12.34)		12,997 (52.37)	2,506 (19.46)	
Not employed	5,202 (24.92)	1,333 (25.36)		11,987 (47.63)	3,209 (26.21)	
Health insurance status			0.989			0.836
Insured	13,272 (66.96)	2,086 (15.58)		17,650 (75.04)	4,063 (22.64)	
Uninsured	7,810 (33.04)	1,194 (15.59)		7,334 (24.96)	1,652 (22.79)	
Education			< 0.001			< 0.001
Less than high school	7,688 (30.86)	1,313 (17.20)		9,139 (30.82)	2,421 (26.36)	
High school graduate	4,809 (22.47)	724 (15.54)		5,547 (21.95)	1,240 (22.65)	
Some college/AA degree	4,008 (20.26)	628 (16.05)		5,360 (22.97)	1,205 (22.84)	
≥ College degree	4,577 (26.40)	615 (13.37)		4,938 (24.26)	849 (17.86)	
Poverty status			< 0.001			< 0.001
Below poverty threshold	4,568 (19.47)	995 (22.39)		7,337 (25.87)	2,235 (30.71)	
At or above poverty threshold	16,514 (80.53)	2,285 (13.94)		17,647 (74.13)	3,480 (19.87)	
BMI			< 0.001			< 0.001
Underweight (BMI < 18.5)	122 (0.62)	29 (23.66)		412 (1.80)	85 (23.12)	
Normal weight (BMI ≥ 18.5 & BMI < 25)	5,873 (28.61)	931(15.86)		9,247 (38.93)	1,754 (18.54)	
Overweight (BMI ≥ 25 & BMI < 30)	10,040 (47.19)	1,438 (14.32)		8,466 (33.30)	1,878 (22.28)	
Obese (BMI 30+)	5,047 (23.58)	882 (17.56)		6,859 (25.97)	1,998 (29.35)	
Physical activity			0.237			0.085
Inactive/Insufficient	20,692 (97.95)	3,204 (15.53)		24,635 (98.42)	5,649 (22.75)	
Physically active	390 (2.05)	76 (18.07)		349 (1.58)	66 (18.22)	
Alcohol drinking status			< 0.001			< 0.001
Never	4,262 (19.54)	558 (13.25)		11,096 (41.52)	2,407 (21.74)	
Former	2,816 (12.43)	602 (21.92)		3,032 (11.70)	860 (28.50)	
Current	14,004 (68.03)	2,120 (15.09)		10,856 (46.78)	2,448 (22.05)	
Smoking status			< 0.001			< 0.001
Never	12,975 (60.66)	1,673 (12.92)		20,231 (79.22)	4,240 (20.71)	
Former	4,715 (23.20)	845 (17.83)		2,869 (12.98)	803 (27.20)	
Current	3,392 (16.14)	762 (22.38)		1,884 (7.80)	672 (35.15)	
Multiple chronic diseases			< 0.001			< 0.001
None	13,660 (63.70)	1,629 (12.11)		14,624 (58.08)	2,485 (17.16)	
1 to 2 diseases	6,395 (31.24)	1,285 (19.57)		8,787 (35.36)	2,447 (26.87)	
3 or more diseases	1,027 (5.06)	366 (34.65)		1,573 (6.56)	783 (48.93)	

Frequencies are unweighted while percentages are weighted.

**Table 2:** Multivariable logistic regression analysis of psychological distress and its associated factors among male and female immigrants.

	Male	Female
	OR (95% CI)	OR (95% CI)
Race		
White immigrant	Ref	Ref
Black immigrant	0.94 (0.82, 1.10)	0.89 (0.79, 1.01)
Age		
18–25 years old	Ref	Ref
26–34 years old	1.06 (0.87, 1.30)	1.00 (0.85, 1.19)
35–44 years old	1.03 (0.84, 1.27)	1.01 (0.85, 1.20)
45–54 years old	0.95 (0.75, 1.19)	1.05 (0.87, 1.26)
55–64 years old	0.76* (0.60, 0.98)	0.88 (0.73, 1.07)
≥ 65 years old	0.44*** (0.34, 0.57)	0.59*** (0.48, 0.72)
Acculturation		
Less than 10 years	0.89 (0.79, 1.01)	0.96 (0.86, 1.06)
10 years or more	Ref	Ref
Marital status		
Divorced	1.15 (0.96, 1.37)	1.11 (0.96, 1.28)
Widowed	0.88 (0.67, 1.17)	0.98 (0.84, 1.16)
Separated	1.17 (0.94, 1.47)	1.11 (0.95, 1.29)
Married/living with partner	0.83** (0.73, 0.95)	0.70*** (0.62, 0.79)
Single/never married	Ref	Ref
Region of residence		
Northeast	Ref	Ref
North Central/Midwest	1.21* (1.01, 1.46)	1.04 (0.90, 1.20)
South	0.98 (0.85, 1.13)	0.89* (0.80, 0.98)
West	1.12 (0.97, 1.30)	1.08 (0.97, 1.21)
Employment status		
Employed	Ref	Ref
Not employed	2.29*** (2.04, 2.56)	1.33*** (1.22, 1.45)
Health insurance status		
Insured	0.95 (0.84, 1.07)	0.97 (0.88, 1.06)
Uninsured	Ref	Ref
Education		
Less than high school	1.12 (0.97, 1.28)	1.22** (1.07, 1.38)
High school graduate	1.06 (0.92, 1.22)	1.16* (1.02, 1.31)
Some college/AA degree	1.03 (0.89, 1.20)	1.18* (1.04, 1.33)
≥ College degree	Ref	Ref
Poverty status		
Below poverty threshold	Ref	Ref
At or above poverty threshold	0.72*** (0.65, 0.80)	0.71*** (0.65, 0.78)
BMI		
Underweight (BMI < 18.5)	1.42 (0.87, 2.31)	1.28 (0.97, 1.69)
Normal weight (BMI ≥ 18.5 & BMI < 25)	Ref	Ref
Overweight (BMI ≥ 25 & BMI < 30)	0.89* (0.79, 0.99)	1.14* (1.03, 1.26)
Obese (BMI 30 +)	0.97 (0.84, 1.11)	1.37*** (1.24, 1.52)
Physical activity		
Inactive/Insufficient	Ref	Ref
Physically active	1.29 (0.93, 1.77)	0.81 (0.59, 1.11)
Alcohol drinking status		
Never	Ref	Ref
Former	1.44*** (1.22, 1.71)	1.23** (1.10, 1.39)
Current	1.17* (1.02, 1.34)	1.15** (1.05, 1.25)
Smoking status		
Never	Ref	Ref
Former	1.24*** (1.11, 1.39)	1.29*** (1.15, 1.45)
Current	1.71*** (1.51, 1.94)	1.76*** (1.55, 1.99)
Multiple chronic diseases		
None	Ref	Ref
1 to 2 diseases	1.82*** (1.65, 2.02)	1.73*** (1.59, 1.88)
3 or more diseases	3.64*** (2.99, 4.42)	4.19*** (3.65, 4.81)

OR = Odds ratio. 95% CI = 95% confidence interval. Statistical significance at *p < 0.05, **p < 0.01, and ***p < 0.001. Ref = reference.

## Results

### Population characteristics

Of the 46,066 immigrant participants, 21,082 were male, and 24,984 were female (Table 1). Male and female immigrants had similar patterns of sociodemographic, anthropometric, behavioral, and clinical characteristics. Among male immigrants, a higher proportion identified as White (86.72%), aged 35–44 years (23.37%), married/living with a partner (62.11%), employed (75.08%), overweight (47.19%), inactive/insufficiently physically active (97.95%), current alcohol drinkers (68.03%), and never smokers (60.66%). They mostly had health insurance (66.96%), completed less than high school (30.86%), and lived in the US for ≥ 10 years (77.97%). A higher proportion also reported living in the South (37.03%), at or above the poverty threshold (80.53%), and with no chronic diseases (63.70%).

Among female immigrants, the majority identified as White (86.92%), 35–44 years old (22.15%), married/living with a partner (53.78%), employed (52.37%), underweight/normal (40.73%), inactive/insufficiently physically active (98.42%), current alcohol drinkers (46.78%), and never smokers (79.22%). They mostly had health insurance (75.04%), completed less than high school (30.82%), and lived in the US for ≥ 10 years (81.02%). The majority also reported living in the South (35.73%) at or above the poverty threshold (74.13%) with no chronic diseases (58.08%).

### Prevalence of moderate-severe psychological distress within subgroups of male and female immigrants

As shown in Table 1, female immigrants reported higher prevalence of moderate-severe psychological distress (22.68%) compared to male immigrants (15.58%). Within the female immigrant population, the subgroups which reported higher prevalence of moderate-severe psychological distress included individuals who were White (22.98%), aged 55–64 years (26.69%), lived in the US ≥ 10 years (23.38%), separated (30.44%), lived in the West region (24.01%), were unemployed (26.21%), received less than high school education (26.36%), lived below the poverty threshold (30.71%), were obese (29.35%), former alcohol drinkers (28.50%), current smokers (35.15%), and lived with 3 or more chronic diseases (48.93%). Among male immigrants, higher prevalence of moderate-severe psychological distress was observed for those who were White (15.83%), aged ≥ 65 years (18.22%), lived in the US ≥ 10 years (16.01%), separated (21.21%), lived in the North Central/Midwest region (17.51%), unemployed (25.36%), received less than high school education (17.20%), lived below the poverty threshold (22.39%), were obese (17.56%), former alcohol drinkers (21.92%), current smokers (22.38%), and lived with 3 or more chronic diseases (34.65%). All the differences in moderate-severe psychological distress prevalence were statistically significant.

### Interaction between sex and each of the factors

**Fig. 1:**
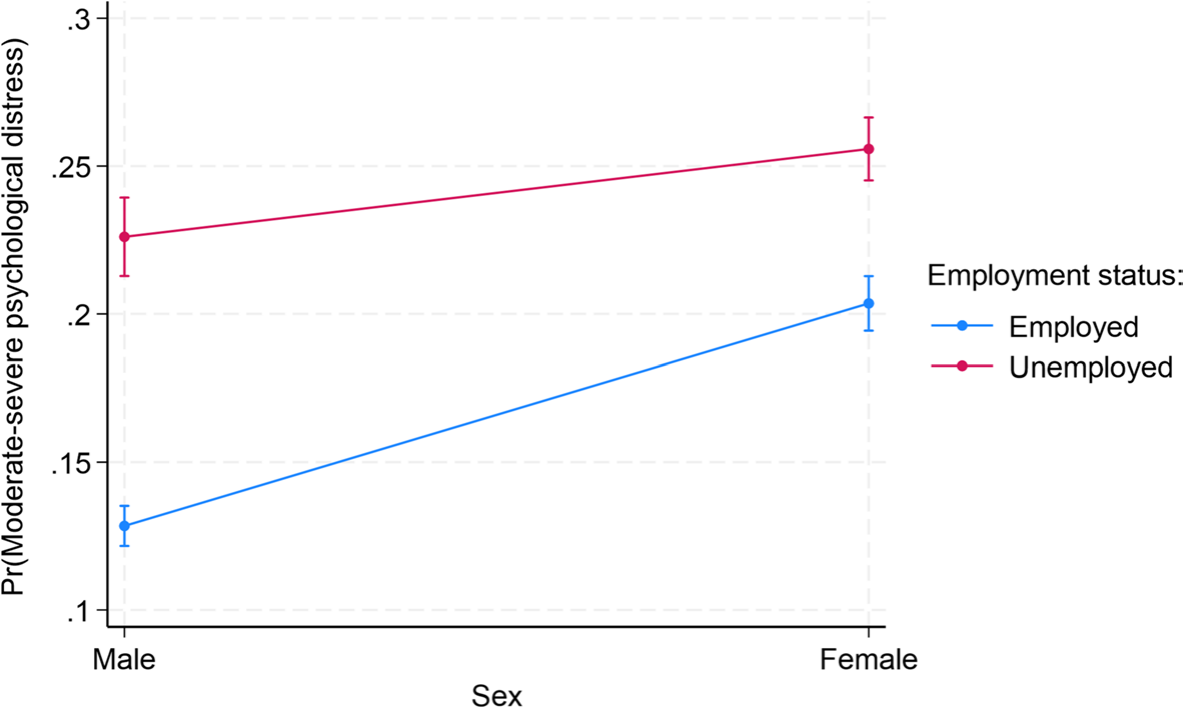
Predictive margins or average predicted probability (with 95% CIs) of moderate-severe psychological distress for each level of interaction of sex and employment status among Black and White adult immigrants in the United States.

**Fig. 2:**
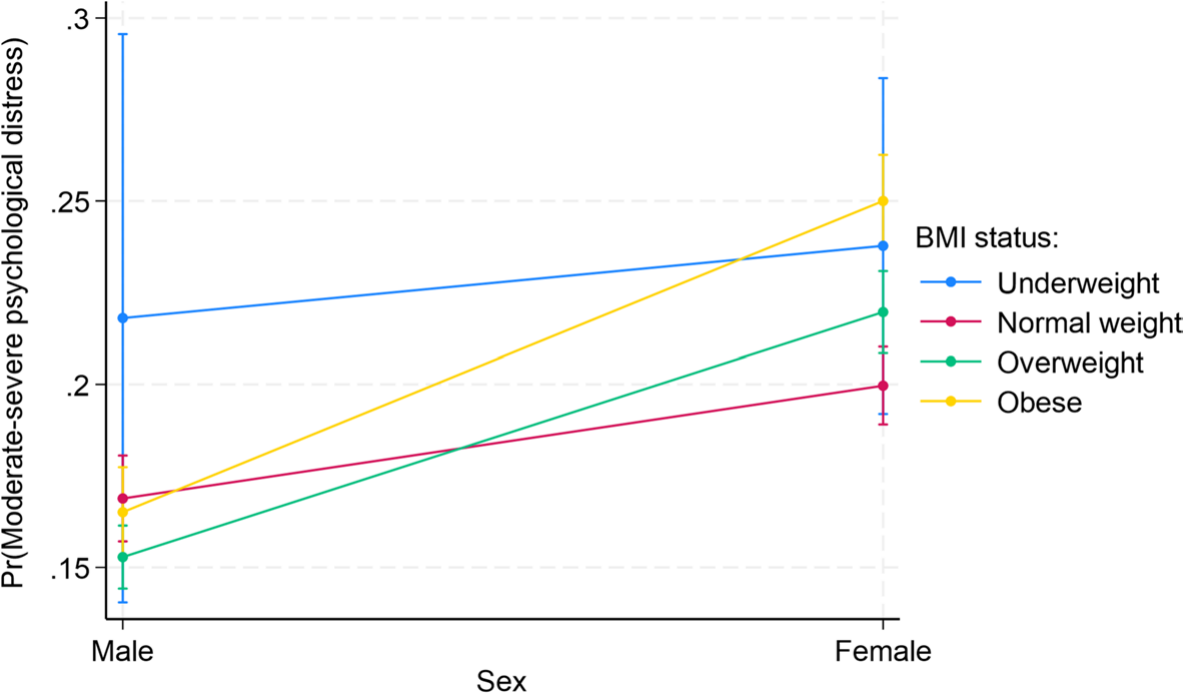
Predictive margins or average predicted probability (with 95% CIs) of moderate-severe psychological distress for each level of interaction of sex and BMI status among Black and White adult immigrants in the United States.

The interactions between sex and each of the factors associated with psychological distress are presented in Supplementary Table S2. Among all the factors, there were statistically significant interactions between only sex and employment status (*p* < 0.001) and BMI status (*p* < 0.001). As shown in Fig. 1, female immigrants who were unemployed (25.58%, *p* < 0.001) had the highest probability of moderate-severe psychological distress, while male immigrants who were employed had the lowest probability (12.84%, *p* < 0.001). For the interaction between sex and BMI status, female immigrants who were obese (25.00%, *p* < 0.001) or underweight (23.78%, *p* < 0.001) similarly had the highest probabilities, while male immigrants who were overweight (15.28%, *p* < 0.001), obese (16.51%, *p* < 0.001), or had normal weight (16.88%, *p* < 0.001) had the lowest probabilities (Fig. 2).

### Factors associated with psychological distress stratified by sex

Table 2 displays the associations between psychological distress and its predictors, stratified by sex. Among male immigrants, higher odds of moderate-severe psychological distress were observed among individuals who were unemployed (OR = 2.29; 95% CI = 2.04, 2.56) compared to employed; former alcohol drinkers (OR = 1.44; 95% CI = 1.22, 1.71) and current alcohol drinkers (OR = 1.17; 95% CI = 1.02, 1.34) compared to never drinkers; and former smokers (OR = 1.24; 95% CI = 1.11, 1.39) and current smokers (OR = 1.72; 95% CI = 1.52, 1.94) compared to never smokers. Higher odds were also observed among those with 1 to 2 (OR = 1.83; 95% CI = 1.65, 2.02) and 3 or more (OR = 3.64; 95% CI = 3.00, 4.42) chronic diseases compared to those with no chronic diseases. Compared to individuals who were living in the US Northeast region, those living in the North Central/Midwest region had higher odds of experiencing moderate-severe psychological distress (OR = 1.21; 95% CI = 1.01, 1.46). Conversely, lower odds of moderate-severe psychological distress were observed among male immigrants who were aged 55–64 years (OR = 0.76; 95% CI = 0.60, 0.98) and ≥ 65 years (OR = 0.44; 95% CI = 0.34, 0.57) relative to 18–25 years; married/living with a partner (OR = 0.83; 95% CI = 0.73, 0.95) compared to single/never married; living at or above the poverty threshold (OR = 0.72; 95% CI = 0.65, 0.80) compared to living below the poverty threshold; and overweight (OR = 0.88; 95% CI = 0.79, 0.99) compared to underweight/normal.

Among female immigrants, higher odds of moderate-severe psychological distress were observed among those who were unemployed (OR = 1.33; 95% CI = 1.22, 1.45) compared to employed; former alcohol drinkers (OR = 1.23; 95% CI = 1.10, 1.39) and current alcohol drinkers (OR = 1.15; 95% CI = 1.05, 1.25) compared to never drinkers; and former smokers (OR = 1.29; 95% CI = 1.15, 1.45) and current smokers (OR = 1.76; 95% CI = 1.56, 2.00) compared to never smokers. Higher odds were also observed among those with 1 to 2 (OR = 1.73; 95% CI = 1.59, 1.88) and 3 or more (OR = 4.19; 95% CI = 3.65, 4.81) chronic diseases compared to those with no chronic diseases. Additionally, compared to individuals who were underweight/normal, those who were overweight (OR = 1.13; 95% CI = 1.02, 1.24) or obese (OR = 1.36; 95% CI = 1.22, 1.50) had higher odds of experiencing moderate-severe psychological distress. Those who had completed less than high school (OR = 1.22; 95% CI = 1.07, 1.38), graduated high school (OR = 1.16; 95% CI = 1.02, 1.31), or had completed some college/associate degree (OR = 1.18; 95% CI = 1.04, 1.33) also had higher odds of experiencing moderate-severe psychological distress compared to those who had completed ≥ college degree. Lower odds of moderate-severe psychological distress were observed among those who were aged ≥ 65 years (OR = 0.59; 95% CI = 0.48, 0.72) relative to 18–25 years; married/living with a partner (OR = 0.70; 95% CI = 0.62, 0.79) compared to single/never married; living at or above the poverty threshold (OR = 0.71; 95% CI = 0.65, 0.78) compared to living below the poverty threshold; and living in the US South region (OR = 0.89; 95% CI = 0.80, 0.98) compared to the Northeast region.

Four of the 13 assessed predictors did not show significant associations with psychological distress across the male and female subgroups. These predictors included race, acculturation, health insurance, and physical activity.

## Discussion

While past research has found that females in general experience higher prevalences of psychological distress compared to males^6,7^, very little research has investigated sex-specific experiences of psychological distress within Black and White immigrant populations. This current study utilized nationally representative sample data to estimate sex differences in psychological distress and discern associations between psychological distress and various anthropometric, behavioral, clinical, and sociodemographic risk factors among male and female immigrant adults.

We found that female immigrants are more likely to experience psychological distress compared to male immigrants, which aligns with past research^6,7,12^. Existing literature on immigrant populations yields mixed results relating to acculturative stressors and psychological distress, with some reporting that females bear greater risk^12,13^. Previous studies have also posited that elevated psychological distress among females in general may be due to factors relating to family, work, or socioenvironmental characteristics^50^. Our analysis further revealed that among all the factors included in our study, only employment status and BMI status significantly moderated the association between sex and psychological distress. Particularly, we found that while male immigrants who were employed had the lowest probability of experiencing psychological distress, female immigrants who were unemployed had the highest probability of experiencing psychological distress. It is possible that employment provides economic, social, and physical activity benefits, especially for males, that can improve mental health and stress levels. Employment provides financial and security support (e.g., consistent income, health or retirement plans, health and wellness programs) and personal and social benefits (e.g., new skills, social connections, sense of purpose and identity, routine) that reduce stress and contribute significantly to overall well-being^51^. Similarly, female immigrants who were obese or underweight were more likely to experience psychological distress compared to their male and female counterparts. The heightened impact of BMI on psychological distress among females could be due to weight bias and menopause-related hormonal changes^52^. While our findings emphasize consideration of employment and BMI in intervention strategies addressing mental health issue among males and females, the underlying reasons for these sex differences remain understudied.

Both sociodemographic (i.e., age), and anthropometric (i.e., BMI) factors assessed in this study were significant indicators of psychological distress within both sexes or the stratified analysis. Specifically, we found that older age (i.e., ≥ 65 vs. 18–25 years) was more significantly associated with a lower likelihood of psychological distress for males than for females. Additionally, males between the ages of 55–64 were also less likely to experience psychological distress. These findings align with past research indicating that older individuals often have lower risks of experiencing psychological distress, a trend that has been attributed to the well-being paradox^53^. However, existing literature is inconsistent in its conclusions regarding the relationship between age, sex, and psychological distress. For instance, while one Australian study found that older females demonstrated lower risks of psychological distress than older males^54^, another reported that higher prevalences of psychological distress among females are maintained, even at older ages^55^. Such findings could be a possible indicator of the extent to which cultural, behavioral, and sociodemographic factors influence gendered experiences across time and space^56^. Being overweight was significantly associated with lower odds of psychological distress among males, whereas being overweight or obese was significantly associated with higher odds of psychological distress among females. Our findings regarding BMI align with past research indicating that gendered beauty standards more negatively impact females’ weight perceptions and mental health compared to males for whom higher BMI has been associated with higher self-esteem^57,58^. Such findings should be taken into consideration in addressing not only physical but also mental health disparities among male and female immigrants.

Behavioral factors such as alcohol drinking status and smoking status were associated with psychological distress, while physical activity was not significantly associated with psychological distress. However, it is important to note the effect sizes or odds ratio estimates showed that being physically active (vs. insufficiently active/inactive) posed greater risk of psychological distress among male immigrants, while being physically active was associated with a lower risk of psychological distress among female immigrants. This finding, coupled with the sex differences in associations between BMI and psychological distress, highlights the importance of conducting longitudinal research to examine the prevalences and impacts of psychological distress predictors. Such studies could better inform future research and the development of public health interventions. Both smoking and alcohol drinking were significantly associated with higher likelihoods of psychological distress among male and female immigrants. Specifically, former and current cigarette smoking had higher effect sizes among female immigrants, while former and current alcohol drinking had higher effect sizes among male immigrants. These associations between smoking, sex, and psychological distress are consistent with existing literature, which has observed that females exhibit more potent associations between smoking and mental health disorders, including depression^59,60^. Past literature has posited that females may exhibit stronger associations between negative affect and smoking and have greater expectations that smoking can reduce negative affect^59^. The finding that alcohol drinking was more strongly associated with psychological distress for male immigrants does not align with past research which concluded that alcohol drinking is more strongly associated with psychological distress among women compared to men^61–63^. In the case of immigrant populations, one study investigating alcohol use among Hispanic immigrants reported that males, who are more likely than their female counterparts to seek employment in the US, are also more likely to engage in substance-use behaviors due to acculturative stress^64^. It should be noted, however, that most existing research examines alcohol drinking behavior as an outcome of psychological distress^65–67^, and not vice versa. More research should seek to understand psychological distress as an outcome of substance-use and lifestyle behaviors to better mitigate associated risk factors.

The findings also revealed a dose–response relationship between the number of chronic conditions and psychological distress for both sexes, with more pronounced impacts among female immigrants. Males with 1 to 2 chronic diseases (vs. no chronic diseases) had higher risks of experiencing psychological distress than their female counterparts, while females with 3 or more chronic diseases had higher risks of experiencing psychological distress than their male counterparts. Similarly, some studies have found that multimorbidity is associated with more severe mental health complications among females when compared to males^68,69^. Notably, one study concluded that women are at nearly double the risk of multimorbidity involving depression compared to men^70^. Among patients with multimorbidity, there is a risk that females are disproportionately affected by suboptimal care^70^ and a decreased sense of self-efficacy^ 68^. Moreover, female immigrants often make more contributions to family care^50,71^, which may exacerbate feelings of inadequacy and further elevate the risk of psychological distress. Given that mental health conditions are harder to diagnose among individuals with chronic diseases^72^, our findings underscore the need for public health measures that address clinical risk factors for psychological distress among immigrant populations.

Region of residence was a significant sociodemographic predictor of psychological distress among both male and female immigrants. Compared to individuals living in the Northeast, females who lived in the Southern region were less likely to experience psychological distress, while males who lived in the North Central/Midwest region had a higher likelihood of experiencing psychological distress. The relatively low risk of psychological distress in the Southern region (for both males and females, though this result was not significant for males) may be due to neighborhood context. Health behaviors of ethnic or racial minority groups can improve when residing in areas with a high density of individuals with similar ethnic or racial identities^73^. Therefore, female immigrants who reside in Southern states such as Texas or Florida (two of the most densely immigrant-populated states in the US)^74^ may benefit from substantial social support that contributes to lower psychological distress^75,76^. Given that male immigrants are more likely to be labor migrants and that Midwestern states have been identified as some of the most exclusionary towards immigrants^77,78^, there is a likelihood that males’ increased exposure to the labor force and workplace discrimination contributes to higher risks of psychological distress in the North Central/Midwest region.

Race was not a significant indicator and there were no significant interactions between race and sex when adjusting for all other predictors. However, both males and females who identified as Black demonstrated lower risks of psychological distress compared to their White counterparts. This finding, though not significant, may indicate the importance of ethno-racial contexts within which immigrants integrate into American society^79^. White immigrants’ increased risks of psychological distress may mirror the higher likelihood of US-born White individuals to experience symptoms of depression or anxiety, since US-born White individuals have been observed as more likely to experience depression and anxiety symptoms compared to US-born Black individuals^80^. However, the association between race, immigration, and psychological distress should be studied further, especially in longitudinal studies, to identify and establish temporal patterns in the relationships for long-term mental health intervention efforts.

Among the other sociodemographic factors assessed, those with significant associations with psychological distress were employment, poverty, marital status, and educational attainment. These significant associations were observed among both males and females, excluding education which was significantly associated with psychological distress among females alone. Unemployment was more strongly associated with psychological distress incidence among males, consistent with past research that has observed unemployment as having more pronounced effects on males’ mental health, possibly due to gendered responsibilities and investments toward family^81^. Regarding poverty, living at or above the poverty threshold was less significantly associated with lower odds of psychological distress for males than for females. Similarly, identifying as married or living with a partner had lower effect sizes for males, a finding which does not align with past research identifying marriage as a weaker protective factor for females^82,83^. Our findings on unemployment, poverty, and marital statuses may be related to remittances. Existing literature reports a pattern of male immigrants being more likely to not only remit but also send larger sums of remittances to their home countries than their female counterparts^84,85^. Such trends may be a function of male immigrants’ roles within their families and their purposes of migration, since males are more likely to be labor migrants and are more likely to have families in their home countries, remitting to spouses among other family members^77,84–86^. Given that financial worries and hardships are linked to the incidence of psychological distress^87–89^, the prevalence of remittance may be an important consideration when assessing stressors and predictors for psychological distress such as employment, poverty and marital statuses within immigrant populations.

Despite males bearing slightly more significant burdens relating to employment, poverty, and marital statuses compared to females, education had no significant impact on the likelihood of males experiencing psychological distress. However, females who had less than high school, a high school diploma, or some college/associate degrees were more likely to experience psychological distress compared to females with a college degree or more. Female immigrants of all education levels may carry even heavier burdens of loneliness and work-to-family conflicts than US-born females due to factors such as acculturative stress^13,14^, higher expectations of family care^71^, and mal-employment/underemployment, which is more common among US-educated female immigrants than male immigrants^90,91^. These dynamics may also help explain our findings that effect sizes for acculturation and health insurance were lower among females, though these results were not statistically significant. Nonetheless, our findings shed light on important differences in anthropometric, behavioral, clinical, and sociodemographic factors and their connections with psychological distress among male and female immigrants.

Given the implications of these findings within growing Black and White immigrant populations^17,18^, more public health research and mental health interventions must be tailored to the needs of male and female immigrants in the US, while more longitudinal or cohort studies are being conducted. For example, these findings suggest potential benefits in sex-specific mental health screening protocols in primary care settings serving immigrant populations^29^. Given the dose–response relationship between chronic diseases and psychological distress, which was especially pronounced among female immigrants, this intervention could be facilitated by integrated care models for addressing chronic disease management alongside mental health^29,92^. Furthermore, targeted workplace mental health programs may be particularly beneficial for male immigrants, given the strong associations between unemployment and psychological distress observed in this group^93^. Additionally, culturally tailored interventions that address the unique stressors faced by immigrant populations, including those related to acculturation and socioeconomic factors, are needed to promote mental health equity in these communities^9,73^.

### Strengths and limitations

This study has several strengths. First, utilizing a large nationally representative dataset from the NHIS, this study was able to achieve findings with generalizability to the broader US Black and White immigrant population. This large sample, pooled over a fourteen-year period, further enhanced statistical power to detect meaningful associations. Third, by employing the K6, a validated and widely used instrument for assessing psychological distress, this study has helped fill an important gap in the literature, as Black and White immigrants remain understudied despite their significant contributions to US population growth.

However, this study also has some limitations. The cross-sectional design of the NHIS precludes causal inference; therefore, the associations observed cannot be interpreted as causal relationships, and the temporal ordering of predictors and psychological distress cannot be entirely established. The reliance on self-reported data may also introduce social desirability bias, potentially leading to underreporting of sensitive behaviors or conditions. Finally, while we examined a comprehensive set of predictors, unmeasured confounders such as discrimination experiences, social support, and immigration-related stressors may influence the observed associations. Future research should assess such factors with greater depth and investigate potential within-group heterogeneity related to country of origin, immigration circumstances, and cultural backgrounds. Such findings could provide more holistic perspectives on minority and immigrant health across racial/ethnic groups, and better inform policies and programs aimed at improving population mental health.

## Conclusions

Overall, this study found that female Black and White immigrants experienced a higher prevalence and odds of psychological distress compared to their male counterparts, with nuanced associations between the predictors and psychological distress that should be considered for future research and interventions. While factors such as old age and BMI were more significantly associated with lower odds of psychological distress among males, several other sociodemographic factors such as employment, poverty, and marital status were more significantly associated with lower odds of psychological distress among females. Education was more significantly associated with higher odds of psychological distress among females only, and region of residence was more significantly associated with higher odds of psychological distress among males alone. Among behavioral factors, smoking was more strongly associated with psychological distress among females, while alcohol drinking was more strongly associated with psychological distress among males. Multiple chronic diseases were associated with elevated psychological distress in both groups, especially for females. These findings contribute to current understandings of immigrant mental health, a topic and a population that remains understudied despite its recent growth, particularly in the context of Black and White US immigrants. As immigrant populations are diverse, more research should examine such sex-related disparities in mental health across various immigrant communities to tailor and promote mental health interventions accordingly. This can ensure a more holistic understanding of immigrant experiences and needs, as well as improved protection and promotion of overall population health.

## Supplementary Information

Below is the link to the electronic supplementary material.

Supplementary Material 1

Supplementary Material 2

## Data Availability

The datasets generated and/or analyzed during the current study are publicly available in the CDC database repository, https://www.cdc.gov/nchs/nhis/data-questionnaires-documentation.htm.
