# Supplementary material for: Sex differences in psychological distress and its risk factors among US adult Black and White immigrants, NHIS 2005–2018
Source: Sci Rep. 2026 Mar 25;16:14920. doi: 10.1038/s41598-026-45360-0 (PMC13168675; doi:10.1038/s41598-026-45360-0)
Supplement: Supplementary file 1 — Supplementary Material 1 [file 41598_2026_45360_MOESM1_ESM.pdf]

```
****************************************************************************************************
*********************
****NOTE: These statistical analysis codes are shared with Scientific Reports editorial team purely or only for their in-house team. The NHIS data is already publicly available. Hence, the shared codes should be treated as confidential with the journal's in-house team.
***Purpose: Psychological Distress and race (Black and White) among Male and Female Adult Immigrants - NHIS from 2005-2018***
***Software: Stata/SE version
18.0                                                                                   ***
***Notes: The datasets were extracted from the IPUMS website: https://nhis.ipums.org/nhis/   ***
****************************************************************************************************
**********************


***Created or pooled the 2005 to 2018 adult datasets to increase the sample size and derive cross-sectional estimates*************
****************************************************************************************************
*************

****Calibrating/adjusting the final sampling weight for the 14 years of datasets to produce average population estimates
***NOTE: For analysis across each year or survey, we will not adjust the weight.
gen weight_adjusted=.
replace weight_adjusted=perweight/14









            ****complete case analysis*****

            ******************************
*****The below variable "complete_cases" generated using egen function rowmiss() is zero only when there are no missing values in an observation for the varlist specified, or implied positive when there are some such missing values.
*****In Stata, zero is treated as false and positive as true when arguments are offered in true-or-false decisions.
egen complete_cases=rowmiss(kessler6_cat acculturation_cat insurance_coverage age_cat5 sex_cat race_status marital_status region_cat4 employment_status education_cat poverty_status bmi_status PhysicalActivity drinking_status smoking_status chronic_diseases birth_place)


************
* Weighted ANALYSIS *
************

*Setting up the survey weight for the weighted analysis
svyset [pweight=weight_adjusted], strata(strata) psu(psu)
svydes

***Use this approach instead. It was used by NHIS (Chceck the NHIS Methodology). Other examples are
located at https://www.stata.com/support/faqs/statistics/standard-error-because-of-stratum/
**This was necessary to address stratum with single sampling unit"
svyset [pweight=weight_adjusted], strata(strata) psu(psu) singleunit(centered)


*******Checking the multicollinearity among the independent variables. Unweighted multicollinearity*
```

```
collin acculturation_cat insurance_coverage age_cat5 sex_cat race_status marital_status region_cat4
employment_status education_cat poverty_status bmi_status PhysicalActivity drinking_status
smoking_status chronic_diseases if age_cat5 >0 & birth_place==1 & race_status<3 & complete_cases==0

*Or for each outcome variable (anxiety and depression). Weighted multicollinearity
logistic kessler6_cat acculturation_cat insurance_coverage age_cat5 sex_cat race_status marital_status
 region_cat4 employment_status education_cat poverty_status bmi_status PhysicalActivity
drinking_status smoking_status chronic_diseases [pweight=weight_adjusted] if age_cat5 >0 & birth_place
==1 & race_status<3 & complete_cases==0

vif, uncentered



                                                    ****TABLE 1. DESCRIPTIVE AND BIVARIATE STATISTICS
OF PSYCHOLOGICAL DISTRESS


                                                    ***Among Male immigrants


****The overall prevalence of psychological distress******
tab kessler6_cat if age_cat5 >0 & sex_cat==0 & birth_place==1 & race_status<3 & complete_cases==0
svy, subpop(if age_cat5 >0 & sex_cat==0 & birth_place==1 & race_status<3 & complete_cases==0): tab
kessler6_cat, format(%12.5g) percent ci

******Race vs. psychological distres******
tab race_status kessler6_cat if age_cat5 >0 & sex_cat==0 & birth_place==1 & race_status<3 &
complete_cases==0
svy, subpop(if age_cat5 >0 & sex_cat==0 & birth_place==1 & race_status<3 & complete_cases==0): tab
race_status kessler6_cat, col format(%12.5g) percent ci
svy, subpop(if age_cat5 >0 & sex_cat==0 & birth_place==1 & race_status<3 & complete_cases==0): tab
race_status kessler6_cat, row format(%12.5g) percent ci

                ***Recoding or labeling race_ethnicity into two categories. This is to compute the
p-value for the chi-square test. We will not save this variable
            recode race_status (1=1 "1 - White only") (2=2 "2 - Black/African American only") (else
=.), gen(race_BlackWhite)
            tab race_BlackWhite

            svy, subpop(if age_cat5 >0 & sex_cat==0 & birth_place==1 & race_status<3 & complete_cases
==0): tab race_BlackWhite kessler6_cat, col format(%12.5g) percent ci
            svy, subpop(if age_cat5 >0 & sex_cat==0 & birth_place==1 & race_status<3 & complete_cases
==0): tab race_BlackWhite kessler6_cat, row format(%12.5g) percent ci

******Age categories vs. psychological distres******
tab age_cat5 kessler6_cat if age_cat5 >0 & sex_cat==0 & birth_place==1 & race_status<3 &
complete_cases==0
svy, subpop(if age_cat5 >0 & sex_cat==0 & birth_place==1 & race_status<3 & complete_cases==0): tab
age_cat5 kessler6_cat, col format(%12.5g) percent ci
svy, subpop(if age_cat5 >0 & sex_cat==0 & birth_place==1 & race_status<3 & complete_cases==0): tab
age_cat5 kessler6_cat, row format(%12.5g) percent ci

****** Number of years spent in the U.S. vs. psychological distres******
tab acculturation_cat kessler6_cat if age_cat5 >0 & sex_cat==0 & birth_place==1 & race_status<3 &
complete_cases==0
svy, subpop(if age_cat5 >0 & sex_cat==0 & birth_place==1 & race_status<3 & complete_cases==0): tab
acculturation_cat kessler6_cat, col format(%12.5g) percent ci
svy, subpop(if age_cat5 >0 & sex_cat==0 & birth_place==1 & race_status<3 & complete_cases==0): tab
acculturation_cat kessler6_cat, row format(%12.5g) percent ci

******Marital status vs. psychological distres******
```

```
tab marital_status kessler6_cat if age_cat5 >0 & sex_cat==0 & birth_place==1 & race_status<3 &
complete_cases==0
svy, subpop(if age_cat5 >0 & sex_cat==0 & birth_place==1 & race_status<3 & complete_cases==0): tab
marital_status kessler6_cat, col format(%12.5g) percent ci
svy, subpop(if age_cat5 >0 & sex_cat==0 & birth_place==1 & race_status<3 & complete_cases==0): tab
marital_status kessler6_cat, row format(%12.5g) percent ci

******Region of residence vs. psychological distres******
tab region_cat4 kessler6_cat if age_cat5 >0 & sex_cat==0 & birth_place==1 & race_status<3 &
complete_cases==0
svy, subpop(if age_cat5 >0 & sex_cat==0 & birth_place==1 & race_status<3 & complete_cases==0): tab
region_cat4 kessler6_cat, col format(%12.5g) percent ci
svy, subpop(if age_cat5 >0 & sex_cat==0 & birth_place==1 & race_status<3 & complete_cases==0): tab
region_cat4 kessler6_cat, row format(%12.5g) percent ci

******Employment status vs. psychological distres******
tab employment_status kessler6_cat if age_cat5 >0 & sex_cat==0 & birth_place==1 & race_status<3 &
complete_cases==0
svy, subpop(if age_cat5 >0 & sex_cat==0 & birth_place==1 & race_status<3 & complete_cases==0): tab
employment_status kessler6_cat, col format(%12.5g) percent ci
svy, subpop(if age_cat5 >0 & sex_cat==0 & birth_place==1 & race_status<3 & complete_cases==0): tab
employment_status kessler6_cat, row format(%12.5g) percent ci

******Health insurance vs. psychological distres******
tab insurance_coverage kessler6_cat if age_cat5 >0 & sex_cat==0 & birth_place==1 & race_status<3 &
complete_cases==0
svy, subpop(if age_cat5 >0 & sex_cat==0 & birth_place==1 & race_status<3 & complete_cases==0): tab
insurance_coverage kessler6_cat, col format(%12.5g) percent ci
svy, subpop(if age_cat5 >0 & sex_cat==0 & birth_place==1 & race_status<3 & complete_cases==0): tab
insurance_coverage kessler6_cat, row format(%12.5g) percent ci

******Educational level vs. psychological distres******
tab education_cat kessler6_cat if age_cat5 >0 & sex_cat==0 & birth_place==1 & race_status<3 &
complete_cases==0
svy, subpop(if age_cat5 >0 & sex_cat==0 & birth_place==1 & race_status<3 & complete_cases==0): tab
education_cat kessler6_cat, col format(%12.5g) percent ci
svy, subpop(if age_cat5 >0 & sex_cat==0 & birth_place==1 & race_status<3 & complete_cases==0): tab
education_cat kessler6_cat, row format(%12.5g) percent ci

******Poverty status vs. psychological distres******
tab poverty_status kessler6_cat if age_cat5 >0 & sex_cat==0 & birth_place==1 & race_status<3 &
complete_cases==0
svy, subpop(if age_cat5 >0 & sex_cat==0 & birth_place==1 & race_status<3 & complete_cases==0): tab
poverty_status kessler6_cat, col format(%12.5g) percent ci
svy, subpop(if age_cat5 >0 & sex_cat==0 & birth_place==1 & race_status<3 & complete_cases==0): tab
poverty_status kessler6_cat, row format(%12.5g) percent ci

******BMI status vs. psychological distres******
tab bmicat kessler6_cat if age_cat5 >0 & sex_cat==0 & birth_place==1 & race_status<3 & complete_cases
==0
svy, subpop(if age_cat5 >0 & sex_cat==0 & birth_place==1 & race_status<3 & complete_cases==0): tab
bmicat kessler6_cat, col format(%12.5g) percent ci
svy, subpop(if age_cat5 >0 & sex_cat==0 & birth_place==1 & race_status<3 & complete_cases==0): tab
bmicat kessler6_cat, row format(%12.5g) percent ci

******Leisure-time physical activity status vs. psychological distres******
tab PhysicalActivity kessler6_cat if age_cat5 >0 & sex_cat==0 & birth_place==1 & race_status<3 &
complete_cases==0
svy, subpop(if age_cat5 >0 & sex_cat==0 & birth_place==1 & race_status<3 & complete_cases==0): tab
PhysicalActivity kessler6_cat, col format(%12.5g) percent ci
svy, subpop(if age_cat5 >0 & sex_cat==0 & birth_place==1 & race_status<3 & complete_cases==0): tab
PhysicalActivity kessler6_cat, row format(%12.5g) percent ci
```

```
******Alcohol use status vs. psychological distres******
tab drinking_status kessler6_cat if age_cat5 >0 & sex_cat==0 & birth_place==1 & race_status<3 &
complete_cases==0
svy, subpop(if age_cat5 >0 & sex_cat==0 & birth_place==1 & race_status<3 & complete_cases==0): tab
drinking_status kessler6_cat, col format(%12.5g) percent ci
svy, subpop(if age_cat5 >0 & sex_cat==0 & birth_place==1 & race_status<3 & complete_cases==0): tab
drinking_status kessler6_cat, row format(%12.5g) percent ci

******Smoking status vs. psychological distres******
tab smoking_status kessler6_cat if age_cat5 >0 & sex_cat==0 & birth_place==1 & race_status<3 &
complete_cases==0
svy, subpop(if age_cat5 >0 & sex_cat==0 & birth_place==1 & race_status<3 & complete_cases==0): tab
smoking_status kessler6_cat, col format(%12.5g) percent ci
svy, subpop(if age_cat5 >0 & sex_cat==0 & birth_place==1 & race_status<3 & complete_cases==0): tab
smoking_status kessler6_cat, row format(%12.5g) percent ci

******Chronic disease status  vs. psychological distres******
tab chronic_diseases kessler6_cat if age_cat5 >0 & sex_cat==0 & birth_place==1 & race_status<3 &
complete_cases==0
svy, subpop(if age_cat5 >0 & sex_cat==0 & birth_place==1 & race_status<3 & complete_cases==0): tab
chronic_diseases kessler6_cat, col format(%12.5g) percent ci
svy, subpop(if age_cat5 >0 & sex_cat==0 & birth_place==1 & race_status<3 & complete_cases==0): tab
chronic_diseases kessler6_cat, row format(%12.5g) percent ci



                                                    ***Among Female immigrants


****The overall prevalence of psychological distress******
tab kessler6_cat if age_cat5 >0 & sex_cat==1 & birth_place==1 & race_status<3 & complete_cases==0
svy, subpop(if age_cat5 >0 & sex_cat==1 & birth_place==1 & race_status<3 & complete_cases==0): tab
kessler6_cat, format(%12.5g) percent ci

******Race vs. psychological distres******
tab race_status kessler6_cat if age_cat5 >0 & sex_cat==1 & birth_place==1 & race_status<3 &
complete_cases==0
svy, subpop(if age_cat5 >0 & sex_cat==1 & birth_place==1 & race_status<3 & complete_cases==0): tab
race_status kessler6_cat, col format(%12.5g) percent ci
svy, subpop(if age_cat5 >0 & sex_cat==1 & birth_place==1 & race_status<3 & complete_cases==0): tab
race_status kessler6_cat, row format(%12.5g) percent ci


            ***Recoding or labeling race_ethnicity into two categories. This is to compute the
p-value for the chi-square test. We will not save this variable
            recode race_status (1=1 "1 - White only") (2=2 "2 - Black/African American only") (else
=.), gen(race_BlackWhite)
            tab race_BlackWhite

            svy, subpop(if age_cat5 >0 & sex_cat==1 & birth_place==1 & race_status<3 & complete_cases
==0): tab race_BlackWhite kessler6_cat, col format(%12.5g) percent ci
            svy, subpop(if age_cat5 >0 & sex_cat==1 & birth_place==1 & race_status<3 & complete_cases
==0): tab race_BlackWhite kessler6_cat, row format(%12.5g) percent ci

******Age categories vs. psychological distres******
tab age_cat5 kessler6_cat if age_cat5 >0 & sex_cat==1 & birth_place==1 & race_status<3 &
complete_cases==0
svy, subpop(if age_cat5 >0 & sex_cat==1 & birth_place==1 & race_status<3 & complete_cases==0): tab
age_cat5 kessler6_cat, col format(%12.5g) percent ci
svy, subpop(if age_cat5 >0 & sex_cat==1 & birth_place==1 & race_status<3 & complete_cases==0): tab
age_cat5 kessler6_cat, row format(%12.5g) percent ci

****** Number of years spent in the U.S. vs. psychological distres******
```

```
tab acculturation_cat kessler6_cat if age_cat5 >0 & sex_cat==1 & birth_place==1 & race_status<3 &
complete_cases==0
svy, subpop(if age_cat5 >0 & sex_cat==1 & birth_place==1 & race_status<3 & complete_cases==0): tab
acculturation_cat kessler6_cat, col format(%12.5g) percent ci
svy, subpop(if age_cat5 >0 & sex_cat==1 & birth_place==1 & race_status<3 & complete_cases==0): tab
acculturation_cat kessler6_cat, row format(%12.5g) percent ci

******Marital status vs. psychological distres******
tab marital_status kessler6_cat if age_cat5 >0 & sex_cat==1 & birth_place==1 & race_status<3 &
complete_cases==0
svy, subpop(if age_cat5 >0 & sex_cat==1 & birth_place==1 & race_status<3 & complete_cases==0): tab
marital_status kessler6_cat, col format(%12.5g) percent ci
svy, subpop(if age_cat5 >0 & sex_cat==1 & birth_place==1 & race_status<3 & complete_cases==0): tab
marital_status kessler6_cat, row format(%12.5g) percent ci

******Region of residence vs. psychological distres******
tab region_cat4 kessler6_cat if age_cat5 >0 & sex_cat==1 & birth_place==1 & race_status<3 &
complete_cases==0
svy, subpop(if age_cat5 >0 & sex_cat==1 & birth_place==1 & race_status<3 & complete_cases==0): tab
region_cat4 kessler6_cat, col format(%12.5g) percent ci
svy, subpop(if age_cat5 >0 & sex_cat==1 & birth_place==1 & race_status<3 & complete_cases==0): tab
region_cat4 kessler6_cat, row format(%12.5g) percent ci

******Employment status vs. psychological distres******
tab employment_status kessler6_cat if age_cat5 >0 & sex_cat==1 & birth_place==1 & race_status<3 &
complete_cases==0
svy, subpop(if age_cat5 >0 & sex_cat==1 & birth_place==1 & race_status<3 & complete_cases==0): tab
employment_status kessler6_cat, col format(%12.5g) percent ci
svy, subpop(if age_cat5 >0 & sex_cat==1 & birth_place==1 & race_status<3 & complete_cases==0): tab
employment_status kessler6_cat, row format(%12.5g) percent ci

******Health insurance vs. psychological distres******
tab insurance_coverage kessler6_cat if age_cat5 >0 & sex_cat==1 & birth_place==1 & race_status<3 &
complete_cases==0
svy, subpop(if age_cat5 >0 & sex_cat==1 & birth_place==1 & race_status<3 & complete_cases==0): tab
insurance_coverage kessler6_cat, col format(%12.5g) percent ci
svy, subpop(if age_cat5 >0 & sex_cat==1 & birth_place==1 & race_status<3 & complete_cases==0): tab
insurance_coverage kessler6_cat, row format(%12.5g) percent ci

******Educational level vs. psychological distres******
tab education_cat kessler6_cat if age_cat5 >0 & sex_cat==1 & birth_place==1 & race_status<3
svy, subpop(if age_cat5 >0 & sex_cat==1 & birth_place==1 & race_status<3): tab education_cat
kessler6_cat, col format(%12.5g) percent ci
svy, subpop(if age_cat5 >0 & sex_cat==1 & birth_place==1 & race_status<3): tab education_cat
kessler6_cat, row format(%12.5g) percent ci

******Poverty status vs. psychological distres******
tab poverty_status kessler6_cat if age_cat5 >0 & sex_cat==1 & birth_place==1 & race_status<3 &
complete_cases==0
svy, subpop(if age_cat5 >0 & sex_cat==1 & birth_place==1 & race_status<3 & complete_cases==0): tab
poverty_status kessler6_cat, col format(%12.5g) percent ci
svy, subpop(if age_cat5 >0 & sex_cat==1 & birth_place==1 & race_status<3 & complete_cases==0): tab
poverty_status kessler6_cat, row format(%12.5g) percent ci

******BMI status vs. psychological distres******
tab bmicat kessler6_cat if age_cat5 >0 & sex_cat==1 & birth_place==1 & race_status<3 & complete_cases
==0
svy, subpop(if age_cat5 >0 & sex_cat==1 & birth_place==1 & race_status<3 & complete_cases==0): tab
bmicat kessler6_cat, col format(%12.5g) percent ci
svy, subpop(if age_cat5 >0 & sex_cat==1 & birth_place==1 & race_status<3 & complete_cases==0): tab
bmicat kessler6_cat, row format(%12.5g) percent ci

******Leisure-time physical activity status vs. psychological distres******
```

```
tab PhysicalActivity kessler6_cat if age_cat5 >0 & sex_cat==1 & birth_place==1 & race_status<3 &
complete_cases==0
svy, subpop(if age_cat5 >0 & sex_cat==1 & birth_place==1 & race_status<3 & complete_cases==0): tab
PhysicalActivity kessler6_cat, col format(%12.5g) percent ci
svy, subpop(if age_cat5 >0 & sex_cat==1 & birth_place==1 & race_status<3 & complete_cases==0): tab
PhysicalActivity kessler6_cat, row format(%12.5g) percent ci

******Alcohol use status vs. psychological distres******
tab drinking_status kessler6_cat if age_cat5 >0 & sex_cat==1 & birth_place==1 & race_status<3 &
complete_cases==0
svy, subpop(if age_cat5 >0 & sex_cat==1 & birth_place==1 & race_status<3 & complete_cases==0): tab
drinking_status kessler6_cat, col format(%12.5g) percent ci
svy, subpop(if age_cat5 >0 & sex_cat==1 & birth_place==1 & race_status<3 & complete_cases==0): tab
drinking_status kessler6_cat, row format(%12.5g) percent ci

******Smoking status vs. psychological distres******
tab smoking_status kessler6_cat if age_cat5 >0 & sex_cat==1 & birth_place==1 & race_status<3 &
complete_cases==0
svy, subpop(if age_cat5 >0 & sex_cat==1 & birth_place==1 & race_status<3 & complete_cases==0): tab
smoking_status kessler6_cat, col format(%12.5g) percent ci
svy, subpop(if age_cat5 >0 & sex_cat==1 & birth_place==1 & race_status<3 & complete_cases==0): tab
smoking_status kessler6_cat, row format(%12.5g) percent ci

******Chronic disease status vs. psychological distres******
tab chronic_diseases kessler6_cat if age_cat5 >0 & sex_cat==1 & birth_place==1 & race_status<3 &
complete_cases==0
svy, subpop(if age_cat5 >0 & sex_cat==1 & birth_place==1 & race_status<3 & complete_cases==0): tab
chronic_diseases kessler6_cat, col format(%12.5g) percent ci
svy, subpop(if age_cat5 >0 & sex_cat==1 & birth_place==1 & race_status<3 & complete_cases==0): tab
chronic_diseases kessler6_cat, row format(%12.5g) percent ci



                                                            **************TABLE 2: WEIGHTED
MULTIVARIABLE LOGISTIC REGRESSION**************************

*******************************************************************************************

                ********Adjusted odds ratios*********
**Model A: sex + age
svy, subpop(if age_cat5 >0 & birth_place==1 & race_status<3 & complete_cases==0): logistic
kessler6_cat ib1.age_cat5 i.sex_cat



********Among Male Immigrants********
svy, subpop(if age_cat5 >0 & sex_cat==0 & birth_place==1 & race_status<3 & complete_cases==0):
logistic kessler6_cat ib1.race_status ib1.acculturation_cat ib1.insurance_coverage ib1.age_cat5 i.
marital_status i.region_cat4 i.employment_status ib3.education_cat ib1.poverty_status ib2.bmicat i.
PhysicalActivity i.drinking_status i.smoking_status i.chronic_diseases


                ********Among Female Immigrants********
svy, subpop(if age_cat5 >0 & sex_cat==1 & birth_place==1 & race_status<3 & complete_cases==0):
logistic kessler6_cat ib1.race_status ib1.acculturation_cat ib1.insurance_coverage ib1.age_cat5 i.
marital_status i.region_cat4 i.employment_status ib3.education_cat ib1.poverty_status ib2.bmicat i.
PhysicalActivity i.drinking_status i.smoking_status i.chronic_diseases
```

```
                                                        **************TABLE 3: TWO-WAY
INTERACTION MODELS***************************

*******************************************************************************************

                ********Adjusted odds ratios*********
**Model A: sex x Race
svy, subpop(if age_cat5 >0 & birth_place==1 & race_status<3 & complete_cases==0): logistic
kessler6_cat ib1.race_status##i.sex_cat ib1.acculturation_cat ib1.insurance_coverage ib1.age_cat5 i.
marital_status i.region_cat4 i.employment_status ib3.education_cat ib1.poverty_status ib2.bmicat i.
PhysicalActivity i.drinking_status i.smoking_status i.chronic_diseases
            ***/* test of overall significant of the interaction. The result is not statistically
significant */
                testparm ib1.race_status#i.sex_cat

**Model B: sex x Age
svy, subpop(if age_cat5 >0 & birth_place==1 & race_status<3 & complete_cases==0): logistic
kessler6_cat ib1.race_status ib1.age_cat5##i.sex_cat ib1.acculturation_cat ib1.insurance_coverage i.
marital_status i.region_cat4 i.employment_status ib3.education_cat ib1.poverty_status ib2.bmicat i.
PhysicalActivity i.drinking_status i.smoking_status i.chronic_diseases
            ***/* test of overall significant of the interaction. The result is not statistically
significant */
                testparm ib1.age_cat5#i.sex_cat

**Model C: sex x Acculturation
svy, subpop(if age_cat5 >0 & birth_place==1 & race_status<3 & complete_cases==0): logistic
kessler6_cat ib1.race_status ib1.age_cat5 ib1.acculturation_cat##i.sex_cat ib1.insurance_coverage i.
marital_status i.region_cat4 i.employment_status ib3.education_cat ib1.poverty_status ib2.bmicat i.
PhysicalActivity i.drinking_status i.smoking_status i.chronic_diseases
            ***/* test of overall significant of the interaction. The result is not statistically
significant */
                testparm ib1.acculturation_cat#i.sex_cat

**Model D: sex x Marital status
svy, subpop(if age_cat5 >0 & birth_place==1 & race_status<3 & complete_cases==0): logistic
kessler6_cat ib1.race_status ib1.age_cat5 ib1.acculturation_cat i.marital_status##i.sex_cat ib1.
insurance_coverage i.region_cat4 i.employment_status ib3.education_cat ib1.poverty_status ib2.bmicat i
.PhysicalActivity i.drinking_status i.smoking_status i.chronic_diseases
            ***/* test of overall significant of the interaction. The result is not statistically
significant */
                testparm i.marital_status#i.sex_cat


**Model E: sex x Region of Residence
svy, subpop(if age_cat5 >0 & birth_place==1 & race_status<3 & complete_cases==0): logistic
kessler6_cat ib1.race_status ib1.age_cat5 ib1.acculturation_cat i.marital_status i.region_cat4##i.
sex_cat ib1.insurance_coverage i.employment_status ib3.education_cat ib1.poverty_status ib2.bmicat i.
PhysicalActivity i.drinking_status i.smoking_status i.chronic_diseases
            ***/* test of overall significant of the interaction. The result is not statistically
significant */
                testparm i.region_cat4#i.sex_cat


**Model F: sex x Employment status
svy, subpop(if age_cat5 >0 & birth_place==1 & race_status<3 & complete_cases==0): logistic
kessler6_cat ib1.race_status ib1.age_cat5 ib1.acculturation_cat i.marital_status i.region_cat4 i.
employment_status##i.sex_cat ib1.insurance_coverage ib3.education_cat ib1.poverty_status ib2.bmicat i.
PhysicalActivity i.drinking_status i.smoking_status i.chronic_diseases
            ***/* test of overall significant of the interaction. The result is statistically
significant */
                testparm i.employment_status#i.sex_cat
```

```
                ***The margins analysis of the interaction effects
        svy, subpop(if age_cat5 >0 & birth_place==1 & race_status<3 & complete_cases==0): logistic
        kessler6_cat ib1.race_status ib1.age_cat5 ib1.acculturation_cat i.marital_status i.region_cat4 i.
        employment_status##i.sex_cat ib1.insurance_coverage ib3.education_cat ib1.poverty_status ib2.bmicat  i.
        PhysicalActivity i.drinking_status i.smoking_status i.chronic_diseases

                ***To get predictive margins or The average predicted probabilities
                margins i.sex_cat#i.employment_status, subpop(if age_cat5 >0 & birth_place==1 &
        race_status<3 & complete_cases==0)

                *Marginsplot
                 marginsplot

        **Model G: sex x Health Insurance status
        svy, subpop(if age_cat5 >0 & birth_place==1 & race_status<3 & complete_cases==0): logistic
        kessler6_cat ib1.race_status ib1.age_cat5 ib1.acculturation_cat i.marital_status i.region_cat4 i.
        employment_status ib1.insurance_coverage##i.sex_cat ib3.education_cat ib1.poverty_status ib2.bmicat  i.
        PhysicalActivity i.drinking_status i.smoking_status i.chronic_diseases
                ***/* test of overall significant of the interaction. The result is not statistically
        significant */
                testparm ib1.insurance_coverage#i.sex_cat


        **Model H: sex x Educational status
        svy, subpop(if age_cat5 >0 & birth_place==1 & race_status<3 & complete_cases==0): logistic
        kessler6_cat ib1.race_status ib1.age_cat5 ib1.acculturation_cat i.marital_status i.region_cat4 i.
        employment_status ib1.insurance_coverage ib3.education_cat##i.sex_cat ib1.poverty_status ib2.bmicat i.
        PhysicalActivity i.drinking_status i.smoking_status i.chronic_diseases
                ***/* test of overall significant of the interaction. The result is not statistically
        significant */
                testparm ib3.education_cat#i.sex_cat


        **Model I: sex x Poverty status
        svy, subpop(if age_cat5 >0 & birth_place==1 & race_status<3 & complete_cases==0): logistic
        kessler6_cat ib1.race_status ib1.age_cat5 ib1.acculturation_cat i.marital_status i.region_cat4 i.
        employment_status ib1.insurance_coverage ib3.education_cat ib1.poverty_status##i.sex_cat ib2.bmicat i.
        PhysicalActivity i.drinking_status i.smoking_status i.chronic_diseases
                ***/* test of overall significant of the interaction. The result is not statistically
        significant */
                testparm ib1.poverty_status#i.sex_cat


        **Model J: sex x BMI status
        svy, subpop(if age_cat5 >0 & birth_place==1 & race_status<3 & complete_cases==0): logistic
        kessler6_cat ib1.race_status ib1.age_cat5 ib1.acculturation_cat i.marital_status i.region_cat4 i.
        employment_status ib1.insurance_coverage ib3.education_cat ib1.poverty_status ib2.bmicat##i.sex_cat  i.
        PhysicalActivity i.drinking_status i.smoking_status i.chronic_diseases
                ***/* test of overall significant of the interaction. The result is statistically
        significant */
                testparm ib2.bmicat#i.sex_cat

                ***The margins analysis of the interaction effects
        svy, subpop(if age_cat5 >0 & birth_place==1 & race_status<3 & complete_cases==0): logistic
        kessler6_cat ib1.race_status ib1.age_cat5 ib1.acculturation_cat i.marital_status i.region_cat4 i.
        employment_status ib1.insurance_coverage ib3.education_cat ib1.poverty_status ib2.bmicat##i.sex_cat  i.
        PhysicalActivity i.drinking_status i.smoking_status i.chronic_diseases

                ***To get predictive margins or The average predicted probabilities
                margins i.sex_cat#ib2.bmicat, subpop(if age_cat5 >0 & birth_place==1 & race_status<3 &
         complete_cases==0)

                *Marginsplot
```

```
                  marginsplot

        **Model K: Sex x Physical Activity status
        svy, subpop(if age_cat5 >0 & birth_place==1 & race_status<3 & complete_cases==0): logistic
        kessler6_cat ib1.race_status ib1.age_cat5 ib1.acculturation_cat i.marital_status i.region_cat4 i.
        employment_status ib1.insurance_coverage ib3.education_cat ib1.poverty_status ib2.bmicat i.
        PhysicalActivity##i.sex_cat i.drinking_status i.smoking_status i.chronic_diseases
                    ***/* test of overall significant of the interaction. The result is not statistically
        significant */
                        testparm i.PhysicalActivity#i.sex_cat


        **Model L: Sex x Alcohol Drinking Status
        svy, subpop(if age_cat5 >0 & birth_place==1 & race_status<3 & complete_cases==0): logistic
        kessler6_cat ib1.race_status ib1.age_cat5 ib1.acculturation_cat i.marital_status i.region_cat4 i.
        employment_status ib1.insurance_coverage ib3.education_cat ib1.poverty_status ib2.bmicat i.
        PhysicalActivity i.drinking_status##i.sex_cat i.smoking_status i.chronic_diseases
                    ***/* test of overall significant of the interaction. The result is not statistically
        significant */
                        testparm i.drinking_status#i.sex_cat


        **Model M: Sex x Smoking Status
        svy, subpop(if age_cat5 >0 & birth_place==1 & race_status<3 & complete_cases==0): logistic
        kessler6_cat ib1.race_status ib1.age_cat5 ib1.acculturation_cat i.marital_status i.region_cat4 i.
        employment_status ib1.insurance_coverage ib3.education_cat ib1.poverty_status ib2.bmicat i.
        PhysicalActivity i.drinking_status i.smoking_status##i.sex_cat i.chronic_diseases
                    ***/* test of overall significant of the interaction. The result is not statistically
        significant */
                        testparm i.smoking_status#i.sex_cat


        **Model M: Sex x Multiple Chronic Diseases
        svy, subpop(if age_cat5 >0 & birth_place==1 & race_status<3 & complete_cases==0): logistic
        kessler6_cat ib1.race_status ib1.age_cat5 ib1.acculturation_cat i.marital_status i.region_cat4 i.
        employment_status ib1.insurance_coverage ib3.education_cat ib1.poverty_status ib2.bmicat i.
        PhysicalActivity i.drinking_status i.smoking_status i.chronic_diseases##i.sex_cat
                    ***/* test of overall significant of the interaction. The result is not statistically
        significant */
                        testparm i.chronic_diseases#i.sex_cat
```
