# Supplementary material for: Sex differences in psychological distress and its risk factors among US adult Black and White immigrants, NHIS 2005–2018
Source: Sci Rep. 2026 Mar 25;16:14920. doi: 10.1038/s41598-026-45360-0 (PMC13168675; doi:10.1038/s41598-026-45360-0)
Supplement: Supplementary file 2 — Supplementary Material 2 [file 41598_2026_45360_MOESM2_ESM.docx]

**SUPPLEMENTARY INFORMATION**

**Supplementary Figure S1.** Flowchart illustrating criteria for psychological distress among male and female adult immigrants’ inclusion and exclusion, NHIS 2005-2018.

Excluded (144,942)

- Non-Black Immigrant
- Non-White Immigrant

Total US Adult Population

N= 925,045

Starting Total Immigrant Population

N= 200,693

Total Included in Analysis

n= 55,751

Male [n= 25,072]; Female [n= 30,679]

Excluded (724,352)

- Non-Immigrant

**Male**

**n= 21,082**

**Female**

**n= 24,984**

**Complete Case Analysis**

**n= 46,066**

Excluded (9,685)

- Missingness
  - Kessler 6 Scale (1,280)
  - Acculturation (810)
  - Health insurance status (185)
  - Marital status (159)
  - Employment status (38)
  - Education (682)
  - Poverty status (5,964)
  - BMI (2,434)
  - Alcohol drinking status (1,012)

**Complete Case Analysis**

**n= 46,066**

**Female**

**n= 24,984**

**Male**

**n= 21,082**

**Supplementary Table S1.** Study variables, NHIS questions, responses, and recode.

| **Variables** | **Question/description** | **Responses** | **Response levels to be analyzed as:** |
| --- | --- | --- | --- |
| **Outcome/dependent variable** | | | |
| Serious psychological distress (SPD): Kessler 6 scale | DURING THE PAST 30 DAYS, how often did you feel…  (1) nervous?  (2) hopeless?  (3) restless or fidgety?  (4) so depressed that nothing could cheer you up?  (5) that everything was an effort?  (6) worthless? | 1= All of the time, 2= Most of the time, 3= Some of the time, 4= A little of the time, 5= None of the time, 6= NIU, 7= Unknown-refused, 8= Unknown-not ascertained, 9= Unknown-don't know | ***For each the item, response levels greater than 4 were set to missing.  ***The response options were reverse coded for the six items to create values from 0 to 4 create scores based on the scale scoring:  0 = None of the time  1 = A little of the time  2 = Some of the time  3 = Most of the time  4 = All of the time  ***The six items were combined to create a composite score ranging from 0-24.    ***Dichotomized the composite score as:  Scores >5 indicating moderate-severe psychological distress, while scores <5 indicate no to mild psychological distress |
| **Independent variables** | | | |
| Race | Self-reported Race (Post-1997 OMB standards):  What race or races [fill1: Do you/Does ALIAS] consider [fill2: yourself/himself/herself] to be? Please select 1 or more of these categories. | 100= White only  200= Black/African American only  300= American Indian/Alaska Native only  400= Asian only  530= Race Group Not Releasable  541= Multiple Race (1999-2018: Including American Indian/Alaska Native) | Recategorize as:  100= White only  200= Black/African American only  300= American Indian/Alaska Native only  400= Asian only  530= Race Group Not Releasable  541= Multiple Race (Including American Indian/Alaska Native) |
| Immigration status | Born in the United States | 11 = No, born in U.S. territory  12 = No, born outside U.S. and U.S. territories  20 = Yes, born in U.S.  96 = NIU  97 = Unknown-refused  98 = Unknown-not ascertained  99 = Unknown-don't know | Non-immigrant: 20 = Yes, born in U.S.  Immigrant: 11/12 = born in U.S. territory or born outside U.S. and U.S. territories  Missing: All other options (96-99) |
| Sex | [Are/Is] [you/person] male or female? | 1 = Male  2= Female | 1 = Male  2= Female |
| **Covariates** | | | |
| Age | What is [fill: your/ALIAS's] age? | Age in years from 0 | 0/17= Under 18  18/25= 18-25  26/34= 26-34  35/44= 35-44  45/54= 45-54  55/64= 55-64  65/85= >=65 |
| Acculturation/length of stay in the US | About how long [fill1: have you/has ALIAS] been in the United States? | 0 = NIU  1= Less than 1 year  2= 1 year to less than 5 years  3= 5 years to less than 10 years  4= 10 years to less than 15 years  5= 15 years or more  8 = Unknown-not ascertained | 1/2/3= Less than 10 years  4/5= 10 years or more  8= Missing |
| Marital status | [Are you/Is ALIAS] now married, widowed, divorced, separated, never married, or living with a partner? | 1= Married, spouse present  2= Married, spouse absent  4= Separated  5= Divorced  6= Widowed 7= Living with partner 8= Never Married 9= Don't know | 1/2/7= Married or living with partner  4= Separated  5= Divorced  6= Widowed 8= Never married or single  9= Missing |
| Region of residence | Region of residence | 1= Northeast  2= North Central/Midwest  3= South  4= West | 1= Northeast  2= North Central/Midwest  3= South  4= West |
| Employment | Employment in past 1 to 2 weeks:  Which of the following [fill: were you/was ALIAS] doing last week? | 0= NIU  111= Working for pay at job/business  112= Working, w/out pay, at job/business  120= With job, but not at work  200= Not employed  220= Not in labor force  997= Unknown-refused  998= Unknown-not ascertained  999= Unknown-don’t know | 111/112/120= Employed  200/220= Unemployed  997/998/999= Missing |
| Education | What is the HIGHEST level of school {person has} completed or the highest degree {person has} received? | 0= NIU  102= Never attended/kindergarten only  104= Grade 1  105= Grade 2  106= Grade 3  107= Grade 4  108= Grade 5  109= Grade 6  110= Grade 7  111= Grade 8  113= Grade 9  114= Grade 10  115= Grade 11  116= 12th grade, no diploma  201= High school graduate  202= GED or equivalent  301= Some college, no degree  302= AA degree: technical/vocational/occupational  303= AA degree: academic program  400= Bachelor's degree (BA,AB,BS,BBA)  501= Master's degree (MA,MS,Med,MBA)  502= Professional (MD,DDS,DVM,JD)  503= Doctoral degree (PhD, EdD)  997= Unknown-refused  998= Unknown-not ascertained  999= Unknown-don’t know | 102-116= Less than High School  201-202= High School graduate  301-303= Technical or Some college  400-503= College or higher  997/998/999= Missing |
| Poverty status | The respondents were also asked about their family’s income relative to the federal poverty threshold (100%, 138%, 200%, 250% and 400%) and consider each family’s size:  (1) Was your total [fill1: family/ ] income from all sources less than [fill2: 250% of poverty threshold] or [fill2: 250% of poverty threshold] or more?  (2) Was your total [fill1: family/ ] income from all sources less than [fill2: 138% of poverty threshold] or [fill2: 138% of poverty threshold] or more?  (3) Was your total [fill1: family/ ] income from all sources less than [fill2: 100% poverty threshold] or [fill2: 100% poverty threshold] or more?  (4) Was your total [fill1: family/ ] income from all sources less than [fill2: 200% of poverty threshold] or [fill2: 200% of poverty threshold] or more?  (5) Was your total [fill1: family/ ] income from all sources less than [fill2: 400% of poverty threshold] or [fill2: 400% of poverty threshold] or more? | 1= Less than [fill2: 250% of poverty threshold] 2= [fill2: 250% of poverty threshold] or more 7= Refused 9= Don't know  1= Less than [fill2: 138% of poverty threshold] 2= [fill2: 138% of poverty threshold] or more 7= Refused 9= Don't know  1= Less than [fill2: 100% of poverty threshold] 2= [fill2: 100% poverty threshold] or more 7= Refused 9= Don't know  1= Less than [fill2: 200% of poverty threshold] 2= [fill2: 200% of poverty threshold] or more 7= Refused 9= Don't know  1= Less than [fill2: 400% of poverty threshold] 2= [fill2: 400% of poverty threshold] or more 7= Refused 9= Don't know | Integrated Public Use Microdata Serie (IPUMS) already recoded the response options as:  1= At or above poverty threshold  2= Below poverty threshold  9= Unk (1997+: incl. Undefined)  We analyzed the variable as:  1= At or above poverty threshold  2= Below poverty threshold  9= Missing |
| Insurance coverage | The National Center for Health Statistics (NCHS) created the health insurance variable (HINOTCOVE) and included in the original NHIS public use data. The variable was created based on responses to a series of questions and editing carried out by NCHS staff to indicate whether the person currently lacks health insurance coverage.  Examples of questions asked:  Are you covered by health insurance or some other kind of health care plan?  What kind of health insurance or health care coverage do you have? | 1= No, has coverage  2= Yes, has no coverage  9= Unknown-don't know | 1= Insured  2= Uninsured  9= Missing |
| Body mass index (BMI) | How tall are you without shoes? In inches  How much do you weigh without shoes? In pounds | BMI was already calculated by NCHS and recoded by IPUMS:  Calculated using the formula:  BMI = (Weight [kg])/(Height [m]) squared)) rounded to 2 decimal places. Conversion values: 1 kilogram (kg) = 2.20462 pounds; 1 meter (m) = 39.37008 inches.  1 = Underweight (BMI < 18.5)  2 = Normal weight (BMI >= 18.5 & BMI < 25)  3 = Overweight (BMI >= 25 & BMI < 30)  4 = Obese (BMI 30+) | 1 = Underweight (BMI < 18.5)  2 = Normal or healthy weight (BMI >= 18.5 & BMI < 25)  3 = Overweight (BMI >= 25 & BMI < 30)  4 = Obese (BMI 30+) |
| Leisure-time physical activity | The questions used to assess leisure-time physical activity can be found in the NHIS questionnaire/codebook below:  <https://ftp.cdc.gov/pub/Health_Statistics/NCHS/Survey_Questionnaires/NHIS/2018/english/qadult.pdf>  or on Integrated Public Use Microdata Serie (IPUMS) website: <https://nhis.ipums.org/nhis-action/variables/group/behavior_pa>  Leisure-time physical activity is based on a series of questions about frequency, duration, and intensity of leisure-time physical activities during the two weeks preceding the interview.  A set of questions asking about frequency and duration of light-moderate and vigorous leisure-time physical activity that lasted at least 10 minutes. | The responses for the respective questions are in time period, in minutes, number of units, and times per week. | Leisure-time physical activity (PA) was created according to the weekly PA duration recommended in the 2018 Health and Human Services Physical Activity Guidelines (https://nhis.ipums.org/nhis/resources/srvydesc2020.pdf).  Leisure-time physical activity was considered sufficient if the participants met the goal of 150 minutes per week of moderate activity, 75 minutes per week of vigorous activity, or an equivalent combination of the two.  Physically active or sufficient PA= if the participants met the goal of 150 minutes per week of moderate activity, 75 minutes per week of vigorous activity, or an equivalent combination of the two.  Inactive/insufficient PA= If the participants did not meet the above goal or requirement.  Sample codes are also provided by NCHS on CDC website:  https://archive.cdc.gov/#/details?url=https://www.cdc.gov/nchs/nhis/physical_activity/pa_recodes.htm |
| Cigarette smoking status | (1) Have you smoked at least 100 cigarettes in your entire life?  (2) Do you NOW smoke cigarettes every day, some days or not at all? | 1= Yes 2= No 7= Refused 9= Don't know  1= Every day 2= Some days 3= Not at all 7= Refused 9= Don't know | Never smoked if question one= 2 (No)  Former smoker if question one is “1= Yes” and question two is “3= Not at all”  Current smoker if question one is “1= Yes” and question two is “1= Every day OR 2= Some days”  7/9= Missing  IPUMS also already created smoking status variable called “SMOKESTATUS2” |
| Alcohol drinking status | (1) In ANY ONE YEAR, have you had at least 12 drinks of any type of alcoholic beverage?  (2) In your ENTIRE LIFE, have you had at least 12 drinks of any type of alcoholic beverage? | 1= Yes 2= No 7= Refused 9= Don't know  1= Yes 2= No 7= Refused 9= Don't know | Lifetime abstainer if participants had less than 12 drinks in lifetime  Former drinker if participants had less than 12 drinks in any one year and no drinks in past year OR had at least 12 drinks in any one year in lifetime but no drinks in past year  Current drinker if participants had 1+ drinks in the past year.  IPUMS also already created alcohol drinking status variable called “ALCSTAT1” |
| Chronic diseases | Asthma: Have you EVER been told by a doctor or other health professional that you had asthma? | 1= Yes 2= No 7= Refused 9= Don't know | We created a multiple chronic disease variable to define three mutually exclusive groups:  0= if the participants had none of the chronic diseases  1-2= if the participants had 1 to 2 chronic conditions  >=3 = if the participants had at least 3 chronic conditions  Response options “7= Refused” and “9= Don't know” |
|  | Stroke: Have you EVER been told by a doctor or other health professional that you had ...A stroke? |  |  |
|  | Emphysema: Have you EVER been told by a doctor or other health professional that you had ...Emphysema? |  |  |
|  | Diabetes or sugar diabetes: Have you EVER been told by a doctor or other health professional that you have diabetes or sugar diabetes? |  |  |
|  | Arthritis: Have you EVER been told by a doctor or other health professional that you have some form of arthritis, rheumatoid arthritis, gout, lupus, or fibromyalgia? |  |  |
|  | Cancer: Have you EVER been told by a doctor or other health professional that you had ...Cancer or a malignancy of any kind? |  |  |
|  | Chronic bronchitis: DURING THE PAST 12 MONTHS, have you been told by a doctor or other health professional that you had ...Chronic bronchitis? |  |  |
|  | Coronary heart disease: Have you EVER been told by a doctor or other health professional that you had...Coronary heart disease? |  |  |
|  | Hepatitis: Have you EVER had hepatitis? |  |  |
|  | Hypertension: Have you EVER been told by a doctor or other health professional that you had ... Hypertension, also called high blood pressure? |  |  |
|  | Kidner disease: DURING THE PAST 12 MONTHS, have you been told by a doctor or other health professional that you had......Weak or failing kidneys? - Do not include kidney stones, bladder infections or incontinence. |  |  |

**Supplementary Table S2.** Interaction between sex and each predictor on moderate-severe psychological distress, adjusting for the rest of the predictors

| **Model A: Sex X Race** | | **Model B: Sex X Age** |  | **Model C: Sex X Acculturation** |  |
| --- | --- | --- | --- | --- | --- |
|  | **OR (95% CI)** |  | **OR (95% CI)** |  | **OR (95% CI)** |
| *Main effects*: |  | *Main effects:* |  | *Main effects:* |  |
| **Race** |  | **Age** |  | **Acculturation** |  |
| Black | 0.92 (0.82, 1.04) | 18-25 years old | Ref | <10 years | 0.95 (0.85, 1.07) |
| White | Ref | 26-34 years old | 1.03 (0.85, 1.24) | >10 years | Ref |
|  |  | 35-44 years old | 1.00 (0.83, 1.20) | **Sex** |  |
| **Sex** |  | 45-54 years old | 0.93 (0.76, 1.14) | Female | 1.53 (1.42, 1.64) |
| Female | 1.51*** (1.41, 1.63) | 55-64 years old | 0.80* (0.64, 0.99) | Male | Ref |
| Male | Ref | ≥65 years old | 0.53*** (0.43, 0.66) | *Interaction*: | F(1, 1118)=0.55, p=0.457 |
|  |  | **Sex** |  |  |  |
| *Interaction*: | F (1, 1118)=0.09, p= 0.764 | Female | 1.41** (1.14, 1.73) |  |  |
|  |  | Male | Ref |  |  |
|  |  | *Interaction effects*: | F(5, 1114)=0.89, p=0.484 |  |  |
| **Model D: Sex X Marital status** |  | **Model E: Sex X Region of Residence** |  | **Model F: Sex X Employment status** |  |
| *Main effects*: |  | *Main effects*: |  | *Main effects*: |  |
| **Marital status** |  | **Region of residence** |  | **Employment status** |  |
| Divorced | 1.06 (0.90, 1.25) | Northeast | Ref | Employed | Ref |
| Widowed | 0.87 (0.66, 1.14) | North Central/Midwest | 1.21* (1.01, 1.45) | Not employed | 2.05*** (1.85, 2.27) |
| Separated | 1.09 (0.88, 1.34) | South | 0.97 (0.84, 1.12) | **Sex** |  |
| Married/living with partner | 0.76*** (0.67, 0.86) | West | 1.11 (0.97, 1.28) | Female | 1.78*** (1.63, 1.95) |
| Single/Never married | Ref | **Sex** |  | Male | Ref |
| **Sex** |  | Female | 1.60*** (1.39, 1.84) | *Interaction*: | F(1, 1118)=38.44, p<0.001 |
| Female | 1.55*** (1.34, 1.80) | Male | Ref |  |  |
| Male | Ref | *Interaction*: | F(3, 1116)=0.75, p=0.521 |  |  |
| *Interaction*: | F(4, 1115)=1.26, p=0.285 |  |  |  |  |
| **Model G: Sex X Health Insurance status** |  | **Model H: Sex X Educational status** |  | **Model I: Sex X Poverty status** |  |
| *Main effects*: |  | *Main effects*: |  | *Main effects*: |  |
| **Health insurance status** |  | **Education** |  | **Poverty status** |  |
| ­Insured | 0.95 (0.85, 1.06) | Less than high school | 1.14* (1.00, 1.29) | Below poverty threshold | Ref |
| Uninsured | Ref | High school graduate | 1.08 (0.94, 1.23) | At or above poverty threshold | 0.67*** (0.60, 0.73) |
| **Sex** |  | Some college/AA degree | 1.06 (0.91, 1.22) | **Sex** |  |
| Female | 1.47*** (1.30, 1.67) | >College degree | Ref | Female | 1.41*** (1.26, 1.59) |
| Male | Ref | **Sex** |  | Male | Ref |
| *Interaction*: | F(1, 1118)=0.26, p=0.610 | Female | 1.44*** (1.24, 1.66) | *Interaction*: | F(1, 1118)=1.90, p=0.168 |
|  |  | Male | Ref |  |  |
|  |  | *Interaction*: | F(3, 1116)=0.36, p=0.785 |  |  |
|  |  |  |  |  |  |
| **Model J: Sex X BMI status** |  | **Model K: Sex X Physical Activity** |  | **Model L: Sex X Alcohol drinking** |  |
| *Main effects*: |  | *Main effects*: |  | *Main effects*: |  |
| **BMI** |  | **Physical activity** |  | **Alcohol drinking status** |  |
| Underweight (BMI<18.5) | 1.40 (0.86, 2.30) | Inactive/Insufficient | Ref | Never | Ref |
| Normal weight (BMI >=18.5 & BMI <25) | Ref | Physically active | 1.27 (0.93, 1.74) | Former | 1.39*** (1.18, 1.64) |
| Overweight (BMI >25 & BMI <30) | 0.88* (0.79, 0.98) | **Sex** |  | Current | 1.12 (0.98, 1.28) |
| Obese (BMI 30+) | 0.97 (0.85, 1.11) | Female | 1.52*** (1.42, 1.63) | **Sex** |  |
| **Sex** |  | Male | Ref | Female | 1.50*** (1.31, 1.71) |
| Female | 1.24*** (1.11, 1.40) | *Interaction*: | F(1, 1118)=3.68, p=0.055 | Male | Ref |
| Male | Ref |  |  | *Interaction*: | F(2, 1117)=1.36, p=0.256 |
| *Interaction:* | F(3, 1116)=6.68, p<0.001 |  |  |  |  |
|  |  |  |  |  |  |
| **Model M: Sex X Smoking status** |  | **Model N: Sex X Multiple chronic diseases** |  |  |  |
| *Main effects*: |  | *Main effects*: |  |  |  |
| **Smoking status** |  | **Multiple chronic diseases** |  |  |  |
| Never | Ref | None | Ref |  |  |
| Former | 1.22*** (1.09, 1.36) | 1 to 2 diseases | 1.79*** (1.63, 1.97) |  |  |
| Current | 1.717*** (1.52, 1.94) | 3 or more diseases | 3.69*** (3.07, 4.43) |  |  |
| **Sex** |  | **Sex** |  |  |  |
| Female | 1.48*** (1.36, 1.61) | Female | 1.50*** (1.36, 1.64) |  |  |
| Male | Ref | Male | Ref |  |  |
| *Interaction:* | F(2, 1117)=0.33, p=0.716 | *Interaction:* | F(2, 1117)=1.00, p=0.367 |  |  |

*OR = Odds ratio. 95% CI = 95% confidence interval. Statistical significance at *p<0.05, **p<0.01, and ***p<0.001. Ref= reference*
